# Hash-Chain Fog/Edge: A Mode-Based Hash-Chain for Secured Mutual Authentication Protocol Using Zero-Knowledge Proofs in Fog/Edge

**DOI:** 10.3390/s22020607

**Published:** 2022-01-13

**Authors:** Mayuresh Sunil Pardeshi, Ruey-Kai Sheu, Shyan-Ming Yuan

**Affiliations:** 1 Electrical Engineering and Computer Science Department (EECS), National Chiao Tung University, Hsinchu 30010, Taiwan; pardeshimayuresh.cs07g@nctu.edu.tw; 2 AI Center, Tunghai University, Taichung 407224, Taiwan; 3 Department of Computer Science, Tunghai University, Taichung 407224, Taiwan; rickysheu@thu.edu.tw; 4 Computer Science Department, National Yang Ming Chiao Tung University, Hsinchu 30010, Taiwan

**Keywords:** security, mutual authentication, fog/edge security, security protocol, Internet of Things (IoT)

## Abstract

Authentication is essential for the prevention of various types of attacks in fog/edge computing. Therefore, a novel mode-based hash chain for secure mutual authentication is necessary to address the Internet of Things (IoT) devices’ vulnerability, as there have been several years of growing concerns regarding their security. Therefore, a novel model is designed that is stronger and effective against any kind of unauthorized attack, as IoT devices’ vulnerability is on the rise due to the mass production of IoT devices (embedded processors, camera, sensors, etc.), which ignore the basic security requirements (passwords, secure communication), making them vulnerable and easily accessible. Furthermore, crackable passwords indicate that the security measures taken are insufficient. As per the recent studies, several applications regarding its requirements are the IoT distributed denial of service attack (IDDOS), micro-cloud, secure university, Secure Industry 4.0, secure government, secure country, etc. The problem statement is formulated as the “design and implementation of dynamically interconnecting fog servers and edge devices using the mode-based hash chain for secure mutual authentication protocol”, which is stated to be an NP-complete problem. The hash-chain fog/edge implementation using timestamps, mode-based hash chaining, the zero-knowledge proof property, a distributed database/blockchain, and cryptography techniques can be utilized to establish the connection of smart devices in large numbers securely. The hash-chain fog/edge uses blockchain for identity management only, which is used to store the public keys in distributed ledger form, and all these keys are immutable. In addition, it has no overhead and is highly secure as it performs fewer calculations and requires minimum infrastructure. Therefore, we designed the hash-chain fog/edge (HCFE) protocol, which provides a novel mutual authentication scheme for effective session key agreement (using ZKP properties) with secure protocol communications. The experiment outcomes proved that the hash-chain fog/edge is more efficient at interconnecting various devices and competed favorably in the benchmark comparison.

## 1. Introduction

In the fog/edge computing architecture, security is crucial for the co-operative use of Internet of Things (IoT) devices [1]. Security can be categorized into the defense lines [2] of preventive, reactive, and tolerance. The preventive measures include cryptography, authentication, and access control. The reactive measures use an intrusion detection system (IDS), whereas tolerance uses replication, redundancy control, and content distribution on disk. Our paper uses authentication for securing fog/edge devices, as prevention is better than the cure. Security is critical in the IoT/fog, as per the research challenges presented in the survey paper “Security and privacy in fog computing: Challenges.” [3] for authentication and key agreement. In this paper, we designed various objectives for IoT devices’ secure inter-connectivity. A mutual authentication protocol within the fog/edge allowed us to authenticate multiple devices with each other safely. IoT device vulnerability is on the rise [4] due to the mass production as per the market requirements and the ignoring of the implementation of security measures within them. Henceforth, this leads to the well-known and one of the strongest attacks, IoT-botnet/DDOS, also known as IDDOS, which exponentially increases the power of distributed denial of service attack (DDOS). The calculable attacks include various traditional protocols, which use a combination of a simple nonce, timestamp, and identities, which are not found to be sufficient, i.e., Kerberos [5], Needham–Schroeder, etc. Therefore, we designed an interactive protocol category, which uses a novel mode-based hash chain for mutual authentication using the properties of zero-knowledge proofs. Furthermore, this public key protocol uses the public keys from the distributed database system, i.e., student ids within the authentication of the national inter-university system. Consider an example of a new student at the university system or visiting a remote university who uses his/her device to login to access the Internet, department facilities, the library catalog, high-performance computing, etc., who is first required to complete the HCFE authentication. Initially, the authorization is completed by the cloud layer, used as a distributed database, then the fog server acts as a verifier and the device as prover to complete the HCFE protocol and obtain the authentication privileges.

### 1.1. Background Knowledge

The background knowledge required for this work includes zero-knowledge proofs, the fog/edge computing architecture, the mutual authentication protocol, and a distributed database system. These can be given in detail as follows:

Zero-knowledge proof is a precise scheme that provides the validity of the genuine verifier to the prover by an efficient interaction without disclosing any sensitive information [6]. The cryptographic protocols can be significantly evaluated by using the ZKP properties such as completeness, soundness, and zero-knowledge. The completeness states that the verifier tag will accept the reader as a legal entity when the validity and zero-knowledge proof of the protocol are possessed by a prover. The soundness assures that the verifier tag will be unaccepted as the reader entity possessed by an invalid prover is illegal, whereas zero-knowledge does not reveal any sensitive information about the shared commitments and the verifier only learns about the statement truthness. The ZKP helps to present compelling proofs of the asserted statements. In other words, if no evidence is provided for the claim, then it is considered to be computationally infeasible to mislead with a non-negligible probability. A replay or modification attack may be applied, but this will be an illegitimate interaction and a non-convincible proof, which is applicable to both parties. Therefore, the crucial part is the interaction within ZKP. The fog is a distributed computing architecture [1] including communication, storage, and control, which is brought closer to all the end users, also known as proximity computing within the cloud-to-things continuum. Even though the term fog is optionally used for the edge, it is broader. The fog can be made relevant when providing creative solutions for the cloud’s shortcomings with either 5G, the IoT, or embedded artificial intelligence. Mutual authentication, also known as two-way authentication, is a process in which multiple entities authenticate each other in a communication link. In the network environment, the server authenticates the client and vice versa. The blockchain [7,8] can be defined as a distributed ledger in which records are not required to be stored on multiple servers to avoid redundancy and can be used for transaction auditing. Furthermore, all the records are immutable, ensuring consistency. As the structure of the blockchain is known, it is quite vulnerable and is well known for its limits on transaction processing.

### 1.2. Motivation

The motivation of this paper can be given as follows: How can we secure all layers of the upcoming fog/edge distributed system including the IoT devices? Authentication for everyone must be provided to keep the communications secure and trusted. Therefore, applying zero-knowledge proof (ZKP) in a distributed system helps to achieve dynamic password-based authentication for all the devices uniquely in every authentication phase. The hash-chain protocol helps to simplify the zero-knowledge proof process and deliver mutual authentication. In the cryptographic community, many cryptographers have been thinking about how to better apply ZKP since Shafi Goldwasser, Silvio Micali, and Charles Rackoff in 1989 applied it in interactive proof systems [5]. Although Schnorr’s identification protocol, honest verifier zero-knowledge, and Fiat–Shamir heuristic attempts were made, most of them were not found to be efficient enough for less calculation on resource-constrained devices (IoT). In this paper, an alternate path, applying zero-knowledge proof by using a simplified system of a challenge–response model from a graph-based interactive transition model is approached. The results proved to be efficient across all the layers of the fog/edge model, and the protocol showed how they can be applied in a better way.

### 1.3. Contribution

The contributions/objectives to the hash-chain fog/edge can be given as follows:

Recently, several mutual authentication systems have used the blockchain distributed ledger, which helps to authenticate devices within the network. Subsequently, solutions have been developed as well, but they are incapable of operating without a third-party system. Thus, there is a need to design a system that can operate without a third-party system. To secure the device by authentication, identities are cross-checked from the distributed database system, which is then mutually authenticated by a novel hash-chain protocol supported by a challenge–response model of zero-knowledge proofs. Eventually, the hash-chain fog/edge objectives can be presented as follows:

- Solving the centralized identity key management problem for authentication: Identity key management is a crucial factor for the authentication system. Authenticating a particular device needs to be first verified by the centralized server. Therefore, all the devices/systems within the fog need to be dependent on the centralized server for authentication. However, the fog also needs to update itself to a centralized server for identity key management, which may face a single-point failure. The hash-chain fog/edge provides distributed identity key management with the help of a distributed database system (DDS). The DDS is a popular model for identity key management;
- Novel algorithm for mutual authentication within the fog/edge: Multiple devices/systems need to be uniquely authenticated based on transitions in the challenge–response model for the hash-chain flow. The proposed model is adaptable and capable of uniquely identifying and authenticating devices/systems by the dynamic challenge–response model. The selection of the transition matrix for the challenge model within the sub-branch of the PRN-based random number tree is performed for every device/system across multiple fogs using several ideas from interactive proof systems;
- Proving dynamic authentication by using zero-knowledge proofs (ZKPs): All the devices/systems within the multiple fogs need to solve the challenge–response model for authentication. The three basic properties of ZKP include completeness, in which the required operations need to be achieved within the specific conditions, soundness, where no cheating devices/systems can succeed in convincing an honest authenticating server, and zero-knowledge, in which the devices/system can never gain complete knowledge about the transition/working secret. These properties help us authenticate the devices/systems, as they possess the part of the knowledge to solve the challenge, which is then verified by the authenticating server;
- Usage of minimum infrastructure and less calculation on the IoT device: The use of minimum infrastructure for authentication within the fog/edge helps us eliminate the ticket-granting server (TGS) and the separate authentication server (AS), other than the fog server and service server.
  Thus, no new system need to be installed within this model, which is a virtual network of fog/edge computing. All the authentication is performed uniquely as we use a pseudo-random number generator (PRNG), which helps us make fewer calculations on resource-constrained IoT devices that are configured with low memory capacity and processing capability.

### 1.4. Applications

Several applications motivated us to develop the hash-chain fog/edge, which is stated as below:

- Secure university: All universities within the country can be interconnected for authentication. Henceforth, university students can effectively use facilities within other universities when visiting. These facilities can help them utilize resources for scientific job processing including high-performance servers, CUDA graphics cards, fog/edge testbeds, etc. They can also take advantage of stored resources, research journals, patents, thesis reports, etc;
- Secure Industry 4.0: In Industry 4.0, all the devices can interconnect with each other and share statistics about sensors, manufacturing devices, demand supply operations, overload management, etc. Furthermore, securing them will help avoid malware attacks, protect their IPR/copyrights, and avoid personal/corporate data breaches, phishing, and ransomware;
- Secure government: In this newly coined term, all the government offices can interconnect securely and can improve security for national-level offices, e.g., National Health Insurance (NHI), which was shut down in a major attack, ransomware protection, protection from real estate, fake documents, employment, lottery, or government impersonation fraud, etc.;
- Secure country: In a secure country, WiFi access points can be secured further for IoT devices including traffic lights, temperature sensors, flood sensors, surveillance cameras, power stations, e.g., the U.K. power grid was brought down by attackers. Several infrastructure automation tasks including roads, railways, transport, watercraft ships, airways, communication systems, etc., can be performed.

### 1.5. Synopsis

The paper plan is given as follows: Section 2 introduces various books and journal, conference, and survey papers used as a reference for designing of hash-chain protocols. Section 3 presents the detailed design of a mode-based hash-chain for secured mutual authentication protocol using zero-knowledge proofs in the fog/edge. The details are presented in the sub-sections on the initialization phase, registration phase, authentication phase, communication phase, and revocation phase.

Section 4 explains the mathematical model lemma for completeness, soundness, and zero-knowledge. Section 5 presents security against various types of attacks, i.e., active attacks, passive attacks, and advanced attacks. Section 6 provides the details of the formal analysis of the protocol regarding message exchange, the idealized protocol, and the final results.

Section 7 demonstrates the system configuration, the performance analysis regarding various results, and benchmark comparisons, followed by the conclusions, acknowledgments, and references.

## 2. Literature

As the current systems are developing to adapt to the fog/edge architecture, the security and privacy aspect has not progressed enough to predict the requirements to ensure its safety [1,3,9]. This closely matches the machine-to-machine (M2M) architecture and smart grids, which have been sufficiently studied.

Table 1 presents the HCFE protocol’s comparison with some recent approaches. Hence, we give a literature survey that is associated with the concerns of the fog: Identity-based authentication solutions are provided using a lightweight equipment certification program, providing an efficiency improvement and reducing the bandwidth consumption for transmission. The proposed work by Deng et al. [10] considered public key management flexibility by avoiding the secure channel to obtain keys with the ease of authentication and the digital signature’s implementation in a distributed environment. Here, the absence of a secure channel for key exchange and high digital signature calculations are unsuitable for a massive fog node-based architecture. A recent survey presented by Mukherjee et al. [3] shared various insights about the current fog/edge security concerns and challenges. It included how to safeguard data against malicious fog node attacks, how to identify malicious insiders/outsiders, how to mutually authenticate new fog/edge users within a network, etc. A detailed survey of optimization on an IoT public key infrastructure by Kelly et al. [11] represents how the IoT faces unique security challenges in resource-constrained environments. The PKI was considered again as a secure environment for such devices, and the use of symmetric keys for communication is still a possible threat. A one-time password with ECC, a two-factor authentication scheme, was approached by Shivraj et al. [12]. The scheme presented here was a hybrid system combining Lamport’s one-time password (OTP) and identity-based elliptical curve cryptography (IBE-ECC). Here, we can obtain genuine algorithms for the new fog system architecture. Octopus, a mutual authentication scheme, was presented by Ibrahim et al. [13]. In this scheme, a new user randomly roaming in the system can mutually authenticate himself/herself using a master secret key, and also, the keys are shared with multiple servers joining the network for smart cards/devices. In this case, repeating a master password is still considered to be unsafe in a large-scale fog/edge network model.

On the computational complexity of combinatorial problems, Diffie et al. [17] presented the problem of the maximum number of requests that can be made simultaneously within a network using the concept of edge-disjoint paths (EDP) or computing the maximum flow, which has been proven to be NP-complete. Ultimately, we wanted to overcome this problem by using cryptographic techniques and mathematical functions that can give high security with as few calculations as possible, to achieve a large amount of mutual authentication within a network containing millions of devices in the least possible time. Authentication is best served by ZKP, as shown by Bruce [18] and Mao [19]. We surveyed various works in the literature to study ZKP applications and the use of mathematical methods within it. A complete ZKP ensures maintaining the three properties of completeness, soundness, and zero-knowledge. ZKP has a wide variety of authentication applications that include field programmable gate arrays (FPGAs), cloud computing, RFID, P2P systems, E-auction, etc. The following survey presents a short note about some well-known methods. FPGA design verification using intellectual property (IP) marks by using the zero-knowledge protocol was presented by Saha et al. [20]. The objective of this work was the trusted proof of embedding desired watermarks within the FPGA. This was achieved by non-disclosure of the details of the mark’s position and the details of the embedded signature string as marks. Multi-cloud storage verification of integrity for data possession by cooperative proving was presented by Zhu et al. [21]. The objectives were to provide an effective solution for the issues in the environment of the distributed cloud for data possession by ensuring transparency in the verification, more security, and better performance. Furthermore, the techniques used here were the homomorphic verifiable response and hash index hierarchy (HIH) in the Hadoop distributed file system. Proof of knowledge (PK) aggregation for the verification of identity by using a multi-factor approach was presented by Bhargav et al. [22]. The objectives within this approach are to provide identification to the requesting party P by a service provider SP by aggregating ZKPK protocols with the challenge of co-gap Diffie–Hellman (co-GDH) group assumptions by using bi-linear maps. Pseudo trust (PT) for P2P using zero-knowledge proofs for authentication was presented by Lu et al. [23]. The objective of this PT is to provide anonymity for authentication by using pseudonym trust management. The challenge is provided by using the APSF protocol and the digital signature standard (DSS) reference, whereby experiments were performed on different desktops using different P2P protocols, Gnutella and KaZaA. RFID system authentication using the zero-knowledge authentication protocol (ZKAP) was presented by Liu et al. [6]. The objectives of this ZKAP support anonymity by using alternative modes integrating multiple access control mechanisms. Verification using partial field pseudonym extraction to achieve better time complexity for the challenge used in the ZKAP for authentication was performed with Feige–Fiat–Shamir intractability for large integer factorization and a discrete logarithm algorithm. Periodic k-times anonymous authentication with the violator’s credential revocation was presented by Lian et al. [24]. This paper highlighted the properties of k-times authentication as a solution for credential revoking and providing ZKP for proving k-times in a zero-knowledge way using the challenge methods of strong RSA assumptions (S-RSAs), decisional Diffie–Hellman assumptions (DDH), and q-Diffie–Hellman inversion assumptions (q-DHI). The vehicle-to-infrastructure (V2I) used in communication for out-of-range distances using group key dissemination was presented by Park et al. [25]. The main contributions of this paper were the ZKP used for the receiver vehicle’s subscription verification, checking the integrity of authenticating process messages, and using a key distribution center (KDC) for the re-keying process by the challenge within this ZKP using the large prime p multi-value strong Diffie–Hellman (p-MVSDH) problem. The combinatorial problems’ computational complexity as presented by Diffie et al. [17] for maximum simultaneous requests for the computing maximum flow or edge-disjoint paths (EDP) within the network problem was proven to be NP-complete. The V2G lightweight mutual authentication protocol using a physical unclonable function was presented by Bansal et al. [14]. They presented a vehicle-to-grid-based mutual authentication by a challenge–response model consisting of MAC, nonce, and user key exchange operations for session key generation. A smart card mutual authentication protocol by using ECC was demonstrated by Kumari et al. [26]. The ECDLP and EC-CDHP method combines a challenge, hash operations, ID, and parameter exchange. A cloud-based e-health D2D mutual authentication protocol was presented by Lopes et al. [27]. The proposed protocol was designed for the 3GPP infrastructure for the exchange of ID, MAC, and hash values based on multiple random values. Remote health monitoring systems using signatures in a mutual authentication protocol was demonstrated by Binu et al. [15]. The mobile device was authenticated in a WLAN by the medical server using a hash of the ID, nonce, and cyclic group parameters. A review article for mutual authentication schemes in the Internet of things is presented by Mbarek et al. [28]. This article consisted of RFID authentication protocols’ analysis and comparison by using tree-based authentication, randomized access control, and third-party authentication protocol. A 5G network authentication key exchange (AKE) protocol for multi-server architecture was demonstrated by Wu et al. [29]. The AKE in this paper overcame the perfect forward secrecy and privilege insider attack using several timestamps and the validation of the session keys. A roaming service, which was a mobile network used for mutual authentication of mobile users for privacy preservation, was presented by Madhusudhan et al. [30]. Securing roaming mobile networks against the impersonation of valid mobile devices with clock synchronization was performed by multiplicative group, modulo, and prime number calculations for public key authentication. Blockchains used for the transfer of parcels between UAVs were presented by Beaman et al. [31]. The UAVs exchange the parcels based on their barcode/RFID/tag identification, which is then authenticated after updating the distributed ledger in the blockchain server based on a smart contract. Authenticating secure device Industrial IoT by using a blockchain was demonstrated by Shen et al. [32]. The devices in the Industrial IoT (IIoT) were secured by using identity-based signatures and ephemeral elliptic curve Diffie–Hellman (ECDHE) key exchange techniques, utilized for the authenticating and key agreement operations. Authentication in distributed systems by incorporating a blockchain was presented by Pande et al. [33].

A two-factor authentication workflow was designed by receiving a one-time password entry and distributed ledger entry by using an ECDH public key within the blockchain. A hybrid blockchain authentication of identity for multiple WSNs was demonstrated by Cui et al. [34]. Local and public blockchains were used for hybrid authentication of the WSN nodes. Blockchain-based distributed data validation and authentication were presented by Nainar et al. [35]. A data-chunk-associated digital signature was added to every part, which was generated by a private key and could be verified by multiple entities using a public key within the blockchain distributed ledger. Blockchain-based authentication using telecom networks for two-factor transactions was presented by Mittal et al. [36]. Instead of depending on a one-time password, a third-party merchant helps directly authenticate via blockchain for payment transactions. The customer credentials are utilized for one-time password generation secured by the private key. Secured identity management using a blockchain was presented by Vimadalal et al. [37]. The digital identity of the user was stored on a permissioned blockchain that could be verified by a passport or driving license, and later for identity authentication, and a blockchain entry for ledger records to keep track of the identity.

The following are the limitation/s that were observed in the above-given literature survey:

- High amount of calculation on resource-constrained devices: As most of the protocols above use high-end mathematical models, for zero-knowledge proofs, they are found to be inefficient for IoT resource-constrained devices;
- Not suitable for authenticating a large number of devices: As the protocols have a high amount of calculation, as well as were not tested with IoT device experiments, they are not assured to have better performance in fog/edge computing;
- No details provided for protocol verification: A protocol is verified and found safe from various attacks when presented with protocol verification (logic of authentication [38]), which was found to be missing in most of the papers.

## 3. Materials and Methods

**Figure 1:**
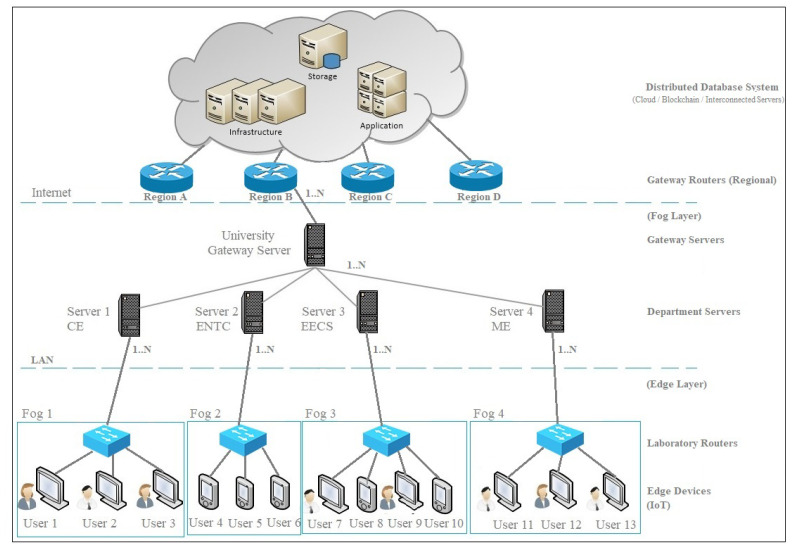
Fog/edge architecture for the university scenario.

In this section, we present various phases of the protocol operations. Each phase demonstrates the detailed design of the hash-chain fog/edge protocol. Figure 1 shows the multiple hierarchical layers involved within the fog/edge computing model. The fog/edge computing model consists of a distributed database layer (top), a fog layer (central), and an edge layer (bottom). In the university scenario, the distributed database layer shows the remote system used for computation, storage, and application, whereas the edge devices are grouped into different fogs based on their proximity for authentication and processing. Each fog can be grouped as the Departments of Civil Engineering (CE), Electronic and Telecommunication Engineering (ENTC), Electrical Engineering and Computer Science (EECS), and Mechanical Engineering (ME). Each fog group contains different types of edge devices, which can be laptops, mobile devices, or IoT devices, which are controlled by their respective fog server’s *FS* in the fog layer. The term fog layer refers to the co-operation between the distributed cloud database for authorization and the fog server as the protocol verifier. The edge layer refers to the processing and exchange of the data from the IoT or user devices as the prover.

### 3.1. Initialization Phase

This is the starting phase for the protocol that defines the initial setup required to achieve appropriate functioning. First, we considered a large organization/university, which is spread in a wide area network, throughout a country as branches, then the following structure was considered. As an organization has branches across the country, then all authentication servers *AS* within an organization representing departments are connected to a common gateway, which acts as a common interface to the external network that should be connected to all other branches’ gateway.

### 3.2. Registration Phase

In the registration phase, every user/client *C* within the organization/university is required to be registered with a unique identity (ID), i.e., for using IoT devices, handheld/portable communicating devices, laptops, etc. The student’s unique ID in the university is uploaded by the administrative authority. More details can be referred from Appendix A.1 Details of the Registration Phase.

**Figure 2:**
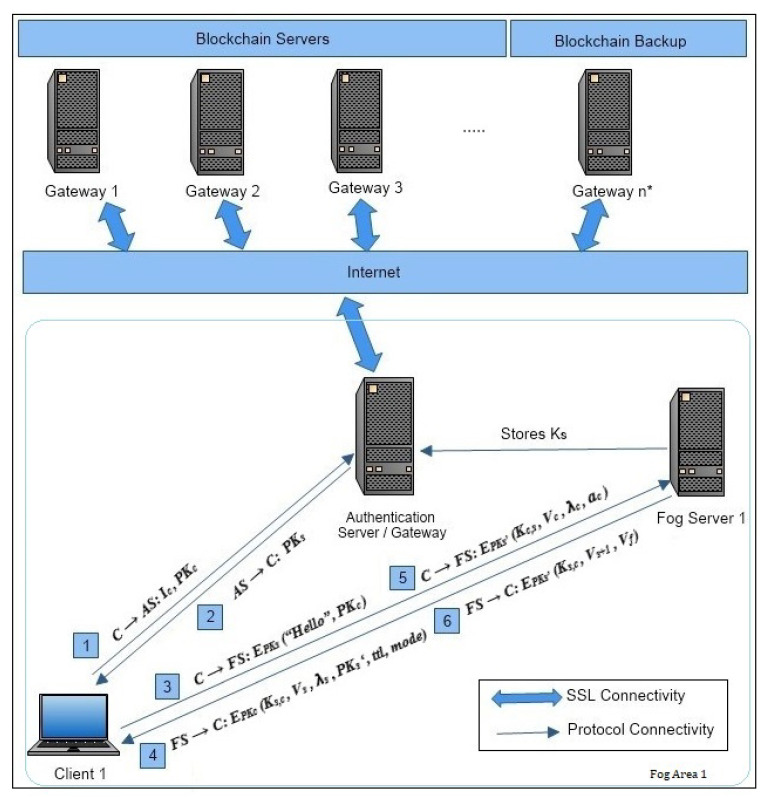
System model for the hash-chain fog/edge protocol.

At the fog layer, the communication is thought to be initialized by all the devices at one point in time or independently. As shown in Figure 2, a couple of *AS*/gateway servers that are most reliable are selected as distributed database servers. Some other *AS* can be reserved to keep a backup of the user IDs in the blockchain/cloud. Distributed database systems are used here for registering all user’s *C* within all organization branches/universities so that a single immutable and distributed ledger copy is maintained in a couple of servers AS across the country to avoid redundancy. The blockchain [39] is known to have the features of trustability, transparency, and reliability. The use of the blockchain proof-of-stake (PoS) [16] has been found to be more suitable as the creator is based on the amount of work performed or random selection. Thus, from each organization branch/university, a single authority will register all user IDs *C* in the blockchain. Henceforth, once the user *C* is registered successfully, then *C* becomes eligible to achieve secure authentication across any branch of the organization/university within the country.

### 3.3. Authentication Phase

**Figure 3:**
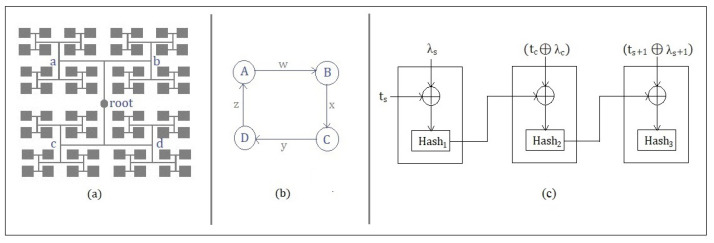
Challenge model. (**a**) Balanced tree with a superfluous sub-branch having 4 × 4 nodes. (**b**) Directed graph transition matrix in each node. (**c**) Hash-chain flow.

In this phase, a user *C* as a registered entity becomes eligible to utilize the facilities within that organization/university, where *C* is physically present. As shown in Figure 3a, the tree structure presents accessibility branches and sub-branches for solving the problem of ZKP for the authentication process. The challenge model contains 16 × 4 tree nodes, generated by a pseudo-random number generator *PRNG*. Each grey/child node contains a unique 2 × 2 matrix that can be accessed in a tree form starting from the root node. The tree traversal simplifies the ease of access to all the parts of the balanced tree.

Each child node has a 2 × 2 matrix given by Figure 3b, which are the transition vales w, x, y, z in the graph. These transition values are the process elements for completing the hash-chain protocol, which is simultaneously registered in the hash-chain flow for the record logs of every client *C*’s authentication. In other words, completing the graph transition of a particular matrix represented as a node within the challenge model tree helps to achieve the authentication successfully between *FS* and *C*. Furthermore, every block has a set of a matrices that is reset every time for a new authentication phase. Figure 3c shows the hash-chain flow, which is created as a part of every hash-chain protocol used for authentication. The hash-chain flow is used to store the transition record logs between the authenticating entities, which can be later referred to for the log auditing process. The root node is created in Step 4 of the hash-chain protocol, consisting of the hash of a unique transition value with its timestamp. The root node’s hash value is then combined (XOR) with the next transition value and a timestamp for generating the second hash ($K_{c,s}$) in Step 5 Similarly, the third hash combines the successive transition value with a timestamp with the previous second hash in Step 6, indicating the completion of the authentication protocol. In that case, the client *C* does not succeed in the first challenge, then C has to complete a new challenge in Mode 1 and later, if un-successful, in Mode 2.

(1) $$\mathrm{Mode}1:D={\left(A\times B\right)}^{-1}\times C^{T}$$

In Equation (1), *FS* sets one of the blocks as in Mode 1. User *C* has to find the block exactly D by calculating the Mode 1 challenge and respond with the values according to the hash-chain protocol.

(2) $$\mathrm{Mode}2:D=A^{T}\times {\left(B\times C\right)}^{-1}$$

Similarly, for Equation (2), *FS* sets one of the blocks as Mode 2. Therefore, the respective user *C* has to find block D and respond to the values according to the hash-chain protocol. In Figure 3, we can see that for every authentication of the corresponding user *C*, a hash-chain is created that helps to maintain the validity of ZKP. This hash-chain process is kept private on the fog server *FS*, and hence, it will not be possible for the user *C* to independently generate it. The first hash1 is taken by the *FS* by including its hash timestamp $t_{s}$ XOR with the transition edge of the selected block matrix $\lambda_{s}$. The previous hash1 value is then XORed with the new set of users *C* (*$t_{c}$ XOR $\lambda_{c}$*) for whom hash2 is generated. hash2, in the same way, is XORed with *FS* ($t_{s+1}$ XOR $\lambda_{s+1}$), for whom hash3 is generated.

The following protocol steps are explained in detail in Figure 2 [38] with the notations:

1. Each client *C* before setting up an authentication first needs to confirm its identity with the authentication server *AS* by sending its identity $I_{c}$ and public key $PK_{c}$.
  $$C\to AS:I_{c}\left|\right|PK_{c};$$
2. The authentication server then responds with the $PK_{s}$ of the local dynamically selected fog server *FS*, only if the client *C* identity and public key are validated from the distributed blockchain.
  $$AS\to C:PK_{s};$$
3. The client *C* then sends a “Hello” message to the local fog server *FS* with its public key $PK_{c}$ encrypted by *FS*’s public key $E_{PKs}$.
  $$C\to FS:E_{PKs}(“\mathrm{Hello}”||PK_{c};)$$
4. The fog server *FS* then considers the request for authentication from user *C* after decrypting the received “Hello” message by the *FS*’s private key $D_{SKs}$. The *FS* then selects one of the branches and blocks randomly from the graph structure to start a ZKP process as the challenge. At first, the hash key $K_{s,c}$ is generated with the hash of $k_{s,c}=H\left(t_{s}⊕\lambda_{s}\right)$. $t_{s}$ represents the current timestamp of the *FS* and $\lambda_{s}$ is the selected random transition value from a block. $V_{s}$ presents the vertex in the graph selected by *FS* and $\lambda_{s}$ as the corresponding transition value. A new session key $PK_{s}^{{}^{\prime }}$ is shared by the *FS*, for encryption of the current authentication messages. *ttl* specifies the validity time of the message and mode as 0 as the default, 1, and 2 for advanced ZKP operations/challenges.
  $$FS\to C:E_{PKc}(K_{s,c}\left|\right|V_{s}\left|\right|\lambda_{s}\left|\right|PK_{s}^{{}^{\prime }}||ttl||mode);$$
5. User *C* then decrypts the message received from the *FS* by his/her private key $D_{SKc}$ and then searches for the vertex $V_{s}$, such that it is reached by the transition $\lambda_{s}$ from the graph structure. Once the user *C* reaches that vertex $V_{s}$, he/she then transits to the next immediate vertex $V_{c}$ and presents the next transition value to the *FS* for the clue. In short, user *C* helps the *FS* reach the alternate vertex within the matrix. Furthermore, a new hash key is generated by the user by $k_{c,s}=H\left(t_{c}⊕\lambda_{c}\right)$, where $t_{c}$ is the current timestamp of the user *C* for sending messages and $\lambda_{c}$ is the clue to the *FS* for the next vertex. The encryption for the new message values is performed by the session key $E_{PKs}^{{}^{\prime }}$ sent by the *FS* in the previous message. $a_{c}$ is used to send client *C*’s IP address to the *FS*.
  $$C\to FS:E_{PKs}^{{}^{\prime }}(K_{c,s}\left|\right|V_{c}\left|\right|\lambda_{c}\left|\right|a_{c});$$
6. Once the *FS* receives the response to a challenge protocol message from the *C*, then decryption is performed using the symmetric session key $D_{SKs}{}^{\prime }$. The *FS* then checks for the vertex flow from the previous challenge given to the *C* and then reaches the new vertex $V_{s+1}$ provided as the clue by the transition $\lambda_{c}$. The *FS* can then calculate the next hash key by using $K_{s,c}$, which is updated by the hash of $k_{s,c}=H\left(t_{s}⊕\lambda_{s}\right)$ with the current ts, and it checks for transition $\lambda_{s}$ to reach the new vertex $V_{s+1}$. The *FS* then embeds the new parameters in the final message with the new vertex $V_{s+1}$ and the final vertex $V_{f}$ of the corresponding matrix, so that the *C* can confirm the matching $V_{s+1}$ and $V_{f}$. After completing a cycle in the matrix sub-graph, the *FS* authenticates *C* as the ZKP is solved and allows the *C* to use updated $K_{s,c}$ for further communication as the final key until the next authentication within the fog.
  $$FS\to C:E_{PKs}^{{}^{\prime }}(K_{s,c}\left|\right|V_{s+1}\left|\right|V_{f})$$

Note: All the cryptographic operations are performed using the Lightweight Encryption System (LES) [40].

### 3.4. Communication Phase

As shown in Figure 2, the architecture is designed such that all client’s *C* are connected to the dynamically selected fog server *FS* within the university/organization. To select the dynamic fog server *FS*, first, client *C* needs to provide his/her identity to the *AS*, which then confirms its validity from the digital identity blockchain. Once the validity is confirmed, it is then provided with the public key of the latest *FS*, which then authenticates all local client’s *C* within that fog. The performance evaluation of the protocol security level considered is less than 400 bit depending on the mode, as the encryption/decryption algorithm we used is 272 bit (LES).

### 3.5. Revocation Phase

All the idle devices or non-responding device’s keys are revoked by the *FS*. To re-initiate authentication, such devices need to solve the challenges using Mode 1 and, if again un-successful, then later using Mode 2. An *FS* maintains a list of revoked client *C*’s keys and allows re-authentication using either the challenge in Mode 1 or Mode 2. The basic purpose of this key revocation phase is to maintain high security within the hash-chain fog/edge protocol.

## 4. Analysis of the Hardness of the Hash-Chain Fog/Edge Zero-Knowledge Protocol (ZKP)

**Theorem** **1.** *Lemma 1 (completeness): If the communicating parties within the protocol AS, FS, and C always follow the complete authentication process, in that case, the approving FS always accepts the C as valid.*

**Proof of Theorem 1.**  In accordance with the authentication protocol steps in the Methodology Section, if the client *C* knows the complete procedure for authentication with the secret of the hash-chain, the key generation, the encryption/decryption algorithm, solving the challenge, and supplying multiple parameters for the next part of the process, then the solution to the challenge received from *C* to *FS*:

$$FS\to C:E_{PKc}(K_{s,c}\left|\right|V_{s}\left|\right|\lambda_{s}\left|\right|PK_{s}^{{}^{\prime }}||ttl||mode).$$

Therefore, $C\to FS:E_{PKs}^{{}^{\prime }}(K_{c,s}\left|\right|V_{c}\left|\right|\lambda_{c}\left|\right|a_{c})$ is considered to be valid, then once the *C* calculates and matches

$$FS\to C:E_{PKs}^{{}^{\prime }}(K_{s,c}\left|\right|V_{s+1}\left|\right|V_{f})$$

then the authentication is known to be successful. □

**Theorem** **2.** *Lemma 2 (soundness): Let us assume it is inefficient to guess and infeasible to calculate several possibilities of the solution to solve the challenge of the hash-chain protocol of Modes 0, 1, and 2 as a malicious user ${\hat{M}}_{u}$ does not possess any of the insufficient knowledge of the FS’s secret. Consider that M takes up the challenge of the FS with impersonating C’s identity and attempts to convince the FS that he/she is a genuine C, then the success probability of ${\hat{M}}_{u}$ is very high.*

**Proof of Theorem 2.**  The process of ZKP is based on the intractability of searching for the solution of Modes 0, 1, and 2. According to number theory, searching for such a solution is equal to calculating matrix combinations. For Mode 0, ${\hat{M}}_{u}$ has to first select the block containing the transition matrix with which the challenge is raised. Note that for each session of authentication for all the fog/edge devices, the random-number-based challenge matrix is updated, and also, the time limit *ttl* must be achieved. To solve Mode 0, ${\hat{M}}_{u}$ has to supply appropriate transition operations to complete the cycle.

$$FS\to C:E_{PKc}(K_{s,c}\left|\right|V_{s}\left|\right|\lambda_{s}\left|\right|PK_{s}^{{}^{\prime }}\left||ttl||mode0\right)$$

For Mode 1, ${\hat{M}}_{u}$ has to achieve the calculation of $D={\left(A\times B\right)}^{-1}\times C^{T}$ by checking all the sub-branches and finding the D that achieves such an expression, then it proceeds to perform the transitions within the time limit.

$$FS\to C:E_{PKc}(K_{s,c}\left|\right|V_{s}\left|\right|\lambda_{s}\left|\right|PK_{s}^{{}^{\prime }}\left||ttl||mode1\right)$$

Similarly, for Mode 2, ${\hat{M}}_{u}$ has to achieve the calculation of $D=A^{T}\times {\left(B\times C\right)}^{-1}$ and complete the transitions for the challenge.

$$FS\to C:E_{PKc}(K_{s,c}\left|\right|V_{s}\left|\right|\lambda_{s}\left|\right|PK_{s}^{{}^{\prime }}\left||ttl||mode2\right)$$

□

**Theorem** **3.** *Lemma 3 (zero-knowledge): The hash-chain fog/edge is a ZKP protocol.*

**Proof of Theorem 3.**  In the hash-chain fog/edge, no clue or information can be revealed about the first key $K_{s,c}$ generation for the hash-chain by the *FS*. As the *FS* can generate and access the challenging matrix in an unspecified time, so the start of $K_{s,c}$ is unknown and hard to guess with several transitions, and the node number is very high. Therefore, it conveys that *M* is not able to guess any possible combination with any of the modes of the challenge in ZKP. Henceforth, this means the hash-chain fog/edge is a ZKP protocol [23]. □

In summary, hash-chain succeeds in completing the ZKP protocol for authentication among the *AS, FS*, and *C*, thus providing effective defense from impersonation attack ${\hat{M}}_{u}$. Section 5 discusses the hash-chain fog/edge’s security [6].

## 5. Hash-Chain Fog/Edge Security

The hash-chain can prevent various security attacks that can be given as the category, attack type, and severity level, 1, 2, and 3. More details about the attack categories is given in Appendix A.2.

### 5.1. Active Attacks

#### 5.1.1. Spoofing

- Severity Level 1:
*${\hat{M}}_{c}$ → AS:* *${\hat{M}}_{Ic}$ || ${\hat{M}}_{PKc}$
*
*AS ↛ ${\hat{M}}_{Ic}$*
  AS:AS has no identity ${\hat{M}}_{Ic}$ with ${\hat{M}}_{PKc}$ in the blockchain. Therefore, the protocol terminates;
- Severity Level 2:
  In this case, *${\hat{M}}_{c}$ → AS:${\hat{M}}_{Ic}$||${\hat{M}}_{PKc}$*
  *AS* →*${\hat{M}}_{c}$: $PK_{s}$*
  *${\hat{M}}_{c}$ ↛ FS: $E_{PKs}$* (“Hello”)
  *AS*: encryption and packet format error. Therefore, the protocol terminates;
- Severity Level 3:
  In this case, ${\hat{M}}_{c}$ → AS:${\hat{M}}_{Ic}$||${\hat{M}}_{PKc}$

$AS\to {\hat{M}}_{c}:PK_{s}$

${\hat{M}}_{c}\to FS:E_{PKs}(‘‘Hello^{\prime \prime }\left|\right|{\hat{M}}_{PKc})$

  $FS\to {\hat{M}}_{c}:E_{PKc}(K_{s,c}\left|\right|V_{s}\left|\right|\lambda_{s}\left|\right|PK_{s}^{{}^{\prime }}\left||ttl||mode0\right)$.
  ${\hat{M}}_{c}$: cannot solve the ZKP challenge in the required time *ttl* for the default mode. Therefore, the protocol terminates.

#### 5.1.2. Modification

- Severity Level 1:
*${\hat{M}}_{c}$ → AS:* *${\hat{M}}_{Ic}$ || ${\hat{M}}_{PKc}$
*
  *AS* → *${\hat{M}}_{c}$: $PK_{s}$*
  *${\hat{M}}_{c}$ → FS: $E_{PKs}$* (“Hello” ||${\hat{M}}_{PKc}$)

$FS\to {\hat{M}}_{c}:E_{PKc}(K_{s,c}\left|\right|V_{s}\left|\right|\lambda_{s}\left|\right|PK_{s}^{{}^{\prime }}\left||ttl||mode=0\right)$

${\hat{M}}_{c}↛FS:E_{PKs}^{{}^{\prime }}(K_{c,s}^{{}^{\prime }}\left|\right|V_{c}^{{}^{\prime }}\left|\right|\lambda_{c}^{{}^{\prime }}\left|\right|a_{c}^{{}^{\prime }})$

  ${\hat{M}}_{c}$: cannot use the modified values from other packets to solve the ZKP challenge in default mode. Therefore, the protocol terminates;
- Severity Level 2:
  After the first unsuccessful attempt, the FS will enter the ZKP challenge.
  Mode 1: *${\hat{M}}_{c}$ → AS:${\hat{M}}_{Ic}$||${\hat{M}}_{PKc}$*
  *AS* → *${\hat{M}}_{c}$: $PK_{s}$*
  *${\hat{M}}_{c}$ → FS: $E_{PKs}$* (“Hello” ||${\hat{M}}_{PKc}$)
  $FS\to {\hat{M}}_{c}:E_{PKc}(K_{s,c}\left|\right|V_{s}\left|\right|\lambda_{s}\left|\right|PK_{s}^{{}^{\prime }}\left||ttl||mode=1\right)$.

${\hat{M}}_{c}↛FS:E_{PKs}^{{}^{\prime }}(K_{c,s}^{{}^{\prime }}\left|\right|V_{c}^{{}^{\prime }}\left|\right|\lambda_{c}^{{}^{\prime }}\left|\right|a_{c}^{{}^{\prime }})$

  ${\hat{M}}_{c}$: cannot solve the ZKP challenge in mode 1. Therefore, the protocol terminates;
- Severity Level 3:
  After the second unsuccessful attempt, the FS will enter the ZKP challenge.
  Mode 2: *${\hat{M}}_{c}$ → AS:${\hat{M}}_{Ic}$||${\hat{M}}_{PKc}$*
  *AS* →*${\hat{M}}_{c}$: $PK_{s}$*
  *${\hat{M}}_{c}$ → FS: $E_{PKs}$* (“Hello” ||${\hat{M}}_{PKc}$)
  $FS\to {\hat{M}}_{c}:E_{PKc}(K_{s,c}\left|\right|V_{s}\left|\right|\lambda_{s}\left|\right|PK_{s}^{{}^{\prime }}\left||ttl||mode=2\right)$.

${\hat{M}}_{c}↛FS:E_{PKs}^{{}^{\prime }}(K_{c,s}^{{}^{\prime }}\left|\right|V_{c}^{{}^{\prime }}\left|\right|\lambda_{c}^{{}^{\prime }}\left|\right|a_{c}^{{}^{\prime }})$

  ${\hat{M}}_{c}$: cannot solve the ZKP challenge in mode 2. Therefore, the protocol terminates.

#### 5.1.3. Sinkhole

- Severity Level 1:
*${\hat{M}}_{c}$ → AS:* *${\hat{M}}_{Ic}$ || ${\hat{M}}_{PKc}$
*
  *AS* →*${\hat{M}}_{c}$: $PK_{s}$*
  *${\hat{M}}_{c}$ → FS: $E_{PKs}$* (“Hello” ||${\hat{M}}_{PKc}$)
  $FS\to {\hat{M}}_{c}:E_{PKc}(K_{s,c}\left|\right|V_{s}\left|\right|\lambda_{s}\left|\right|PK_{s}^{{}^{\prime }}\left||ttl||mode=0\right)$.

${\hat{M}}_{c}↛FS_{1}:E_{PKs}^{{}^{\prime }}(K_{c,s}^{{}^{\prime }}\left|\right|V_{c}^{{}^{\prime }}\left|\right|\lambda_{c}^{{}^{\prime }}\left|\right|a_{c}^{{}^{\prime }})$

  ${\hat{M}}_{c}$: here, the selective modification will not work because of the SHA-256 bit hash used in $K_{c,s}^{{}^{\prime }}$. Therefore, the protocol terminates;
- Severity Level 2:
*${\hat{M}}_{c}$ → AS:* *${\hat{M}}_{Ic}$ || ${\hat{M}}_{PKc}$
*
  *AS* →*${\hat{M}}_{c}$: $PK_{s}$*
  *${\hat{M}}_{c}$ → FS: $E_{PKs}$* (“Hello” ||${\hat{M}}_{PKc}$)
  $FS\to {\hat{M}}_{c}:E_{PKc}(K_{s,c}\left|\right|V_{s}\left|\right|\lambda_{s}\left|\right|PK_{s}^{{}^{\prime }}\left||ttl||mode=0\right)$.

${\hat{M}}_{c}↛FS_{1}:E_{PKs}^{{}^{\prime }}(K_{c,s}^{{}^{\prime }}\left|\right|V_{c}^{{}^{\prime }}\left|\right|\lambda_{c}^{{}^{\prime }}\left|\right|a_{c}^{{}^{\prime }})$

  ${\hat{M}}_{c}$: the use of the $K_{c,s}^{{}^{\prime }}$ hash-chain will be inconsistent with the defined flow. Therefore, the protocol terminates;
- Severity Level 3:
*${\hat{M}}_{c}$ → AS:* *${\hat{M}}_{Ic}$ || ${\hat{M}}_{PKc}$
*
  *AS* →*${\hat{M}}_{c}$: $PK_{s}$*
  *${\hat{M}}_{c}$ → FS: $E_{PKs}$* (“Hello” ||${\hat{M}}_{PKc}$)
  $FS\to {\hat{M}}_{c}:E_{PKc}(K_{s,c}\left|\right|V_{s}\left|\right|\lambda_{s}\left|\right|PK_{s}^{{}^{\prime }}\left||ttl||mode=0\right)$.

${\hat{M}}_{c}↛FS_{n}:E_{PKs}^{{}^{\prime }}(K_{c,s}^{{}^{\prime }}\left|\right|V_{c}^{{}^{\prime }}\left|\right|\lambda_{c}^{{}^{\prime }}\left|\right|a_{c}^{{}^{\prime }})$

  ${\hat{M}}_{c}$: cannot forward selective modified SHA-256 bit hash used in $K_{c,s}^{{}^{\prime }}$ in any other fog. Therefore, the protocol terminates.

### 5.2. Passive Attacks

#### 5.2.1. Eavesdropping (Man-in-the-Middle)

- Severity Level 1:
*${\hat{M}}_{c}$ → AS:* *${\hat{M}}_{Ic}$ || ${\hat{M}}_{PKc}$
*
  *AS* →*${\hat{M}}_{c}$: $PK_{s}$*
  *${\hat{M}}_{c}$ ↛ FS: $E_{PKs}$* (“Hello”)
  ${\hat{M}}_{c}$: cannot apply the required encryption type and specified message format of *AS*. Therefore, the protocol terminates;
- Severity Level 2:
*${\hat{M}}_{c}$ → AS:* *${\hat{M}}_{Ic}$ || ${\hat{M}}_{PKc}$
*
  *AS* →*${\hat{M}}_{c}$: $PK_{s}$*
  *${\hat{M}}_{c}$ → FS: $E_{PKs}$* (“Hello” ||${\hat{M}}_{PKc}$)

$FS\to {\hat{M}}_{c}:E_{PKc}(K_{s,c}\left|\right|V_{s}\left|\right|\lambda_{s}\left|\right|PK_{s}^{{}^{\prime }}\left||ttl||mode=0\right)$

  ${\hat{M}}_{c}$: cannot solve the ZKP challenge in the required time *ttl* of default mode. Therefore, the protocol terminates;
- Severity Level 3:
  After one unsuccessful attempt:
  Mode 1: *${\hat{M}}_{c}$ → AS:${\hat{M}}_{Ic}$||${\hat{M}}_{PKc}$*
  *AS* →*${\hat{M}}_{c}$: $PK_{s}$*
  *${\hat{M}}_{c}$ → FS: $E_{PKs}$* (“Hello” ||${\hat{M}}_{PKc}$)

$FS\to {\hat{M}}_{c}:E_{PKc}(K_{s,c}\left|\right|V_{s}\left|\right|\lambda_{s}\left|\right|PK_{s}^{{}^{\prime }}\left||ttl||mode=1\right)$

${\hat{M}}_{c}↛FS:E_{PKs}^{{}^{\prime }}(K_{c,s}^{{}^{\prime }}\left|\right|V_{c}^{{}^{\prime }}\left|\right|\lambda_{c}^{{}^{\prime }}\left|\right|a_{c}^{{}^{\prime }})$

  ${\hat{M}}_{c}$: cannot solve the ZKP challenge in mode 1. Therefore, the protocol terminates.
  After the second unsuccessful attempt:
  Mode 2: *${\hat{M}}_{c}$ → AS:${\hat{M}}_{Ic}$||${\hat{M}}_{PKc}$*
  *AS* →*${\hat{M}}_{c}$: $PK_{s}$*
  *${\hat{M}}_{c}$ → FS: $E_{PKs}$* (“Hello” ||${\hat{M}}_{PKc}$)

$FS\to {\hat{M}}_{c}:E_{PKc}(K_{s,c}\left|\right|V_{s}\left|\right|\lambda_{s}\left|\right|PK_{s}^{{}^{\prime }}\left||ttl||mode=2\right)$

${\hat{M}}_{c}↛FS:E_{PKs}^{{}^{\prime }}(K_{c,s}^{{}^{\prime }}\left|\right|V_{c}^{{}^{\prime }}\left|\right|\lambda_{c}^{{}^{\prime }}\left|\right|a_{c}^{{}^{\prime }})$

  ${\hat{M}}_{c}$: cannot solve the ZKP challenge in mode 2. Therefore, the protocol terminates.

#### 5.2.2. Monitoring

- Severity Level 1:
*${\hat{M}}_{c}$ → AS:* *${\hat{M}}_{Ic}$ || ${\hat{M}}_{PKc}$
*
*AS ↛ ${\hat{M}}_{Ic}$*
  *AS:* cannot recognize ${\hat{M}}_{Ic}$ with ${\hat{M}}_{PKc}$ in the blockchain. Therefore, the protocol terminates;
- Severity Level 2:
*${\hat{M}}_{c}$ → AS:* *${\hat{M}}_{Ic}$ || ${\hat{M}}_{PKc}$
*
  *AS* →*${\hat{M}}_{c}$: $PK_{s}$*
  *${\hat{M}}_{c}$ → FS: $E_{PKs}$* (“Hello” ||${\hat{M}}_{PKc}$)
  *FS*: cannot recognize the encryption type. Therefore, the protocol terminates;
- Severity Level 3:
*${\hat{M}}_{c}$ → AS:* *${\hat{M}}_{Ic}$ || ${\hat{M}}_{PKc}$
*
  *AS* →*${\hat{M}}_{c}$: $PK_{s}$*
  *${\hat{M}}_{c}$ → FS: $E_{PKs}$* (“Hello” ||${\hat{M}}_{PKc}$)

$FS\to {\hat{M}}_{c}:E_{PKc}(K_{s,c}\left|\right|V_{s}\left|\right|\lambda_{s}\left|\right|PK_{s}^{{}^{\prime }}\left||ttl||mode=0\right)$

  ${\hat{M}}_{c}$: cannot solve the ZKP challenge in the required time *ttl* of default mode. Therefore, the protocol terminates.

### 5.3. Advance Attacks

#### 5.3.1. Replay

- Severity Level 1:
*${\hat{M}}_{c}$ → AS:* *${\hat{M}}_{Ic}$ || ${\hat{M}}_{PKc}$
*
  *AS* →*${\hat{M}}_{c}$: $PK_{s}$*
  *${\hat{M}}_{c}$ ↛ FS: $E_{PKs}$* (“Hello” ||${\hat{M}}_{PKc}$)
  *FS*: replay of the same message at different times, may have a change of the *FS* and does not apply in different *FS*. Therefore, the protocol terminates;
- Severity Level 2:
*${\hat{M}}_{c}$ → AS:* *${\hat{M}}_{Ic}$ || ${\hat{M}}_{PKc}$
*
  *AS* →*${\hat{M}}_{c}$: $PK_{s}$*
  *${\hat{M}}_{c}$ → FS: $E_{PKs}$* (“Hello” ||${\hat{M}}_{PKc}$)

$FS\to {\hat{M}}_{c}:E_{PKc}(K_{s,c}\left|\right|V_{s}\left|\right|\lambda_{s}\left|\right|PK_{s}^{{}^{\prime }}\left||ttl||mode=0\right)$

  ${\hat{M}}_{c}$: cannot solve the ZKP challenge in the required time *ttl* for the default mode. Therefore, the protocol terminates;
- Severity Level 3:
*${\hat{M}}_{c}$ → AS:* *${\hat{M}}_{Ic}$ || ${\hat{M}}_{PKc}$
*
  *AS* →*${\hat{M}}_{c}$: $PK_{s}$*
  *${\hat{M}}_{c}$ → FS: $E_{PKs}$* (“Hello” ||${\hat{M}}_{PKc}$)

$FS\to {\hat{M}}_{c}:E_{PKc}(K_{s,c}\left|\right|V_{s}\left|\right|\lambda_{s}\left|\right|PK_{s}^{{}^{\prime }}\left||ttl||mode=2\right)$

${\hat{M}}_{c}↛FS:E_{PKs}^{{}^{\prime }}(K_{c,s}^{{}^{\prime }}\left|\right|V_{c}^{{}^{\prime }}\left|\right|\lambda_{c}^{{}^{\prime }}\left|\right|a_{c}^{{}^{\prime }})$

  ${\hat{M}}_{c}$: cannot construct a new key $K_{s,c}$ by a valid hash-chain procedure. Therefore, the protocol terminates.

#### 5.3.2. Location Disclosure

- Severity Level 1:
*${\hat{M}}_{c}$ → AS:* *${\hat{M}}_{Ic}$ || ${\hat{M}}_{PKc}$
*
  *AS* →*${\hat{M}}_{c}$: $PK_{s}$*
  *${\hat{M}}_{c}$ → FS: $E_{PKs}$* (“Hello” ||${\hat{M}}_{PKc}$)

$FS\to {\hat{M}}_{c}:E_{PKc}(K_{s,c}\left|\right|V_{s}\left|\right|\lambda_{s}\left|\right|PK_{s}^{{}^{\prime }}\left||ttl||mode=0\right)$

  ${\hat{M}}_{c}$: cannot solve the ZKP challenge in the required time *ttl* for the default mode. Therefore, the protocol terminates;
- Severity Level 2:
  After first unsuccessful attempt, *FS* will enter in the ZKP challenge Mode 1.
*${\hat{M}}_{c}$ → AS:* *${\hat{M}}_{Ic}$ || ${\hat{M}}_{PKc}$
*
  *AS* →*${\hat{M}}_{c}$: $PK_{s}$*
  *${\hat{M}}_{c}$ → FS: $E_{PKs}$* (“Hello” ||${\hat{M}}_{PKc}$)

$FS\to {\hat{M}}_{c}:E_{PKc}(K_{s,c}\left|\right|V_{s}\left|\right|\lambda_{s}\left|\right|PK_{s}^{{}^{\prime }}\left||ttl||mode=1\right)$

${\hat{M}}_{c}↛FS:E_{PKs}^{{}^{\prime }}(K_{c,s}^{{}^{\prime }}\left|\right|V_{c}^{{}^{\prime }}\left|\right|\lambda_{c}^{{}^{\prime }}\left|\right|a_{c}^{{}^{\prime }})$

  ${\hat{M}}_{c}$: cannot solve the ZKP challenge in the required time *ttl* for Mode 1. Therefore, the protocol terminates;
- Severity Level 3:
  After the second unsuccessful attempt, the *FS* will enter the ZKP challenge Mode 2.
*${\hat{M}}_{c}$ → AS:* *${\hat{M}}_{Ic}$ || ${\hat{M}}_{PKc}$
*
  *AS* →*${\hat{M}}_{c}$: $PK_{s}$*
  *${\hat{M}}_{c}$ → FS: $E_{PKs}$* (“Hello” ||${\hat{M}}_{PKc}$)

$FS\to {\hat{M}}_{c}:E_{PKc}(K_{s,c}\left|\right|V_{s}\left|\right|\lambda_{s}\left|\right|PK_{s}^{{}^{\prime }}\left||ttl||mode=2\right)$

${\hat{M}}_{c}↛FS:E_{PKs}^{{}^{\prime }}(K_{c,s}^{{}^{\prime }}\left|\right|V_{c}^{{}^{\prime }}\left|\right|\lambda_{c}^{{}^{\prime }}\left|\right|a_{c}^{{}^{\prime }})$

  ${\hat{M}}_{c}$ cannot solve the ZKP challenge in the required time *ttl* for Mode 2. Therefore, the protocol terminates.

#### 5.3.3. Sybil

- Severity Level 1:
*${\hat{M}}_{c}$ → AS:* *${\hat{M}}_{Ic}$ || ${\hat{M}}_{PKc}$
*
  *AS* →*${\hat{M}}_{c}$: $PK_{s}$*
  *${\hat{M}}_{c}$ ↛ FS: $E_{PKs}$* (“Hello” ||${\hat{M}}_{PKc}$)
  ${\hat{M}}_{c}$: cannot encrypt by the required algorithm for sending a message to the *FS*. Therefore, the protocol terminates;
- Severity Level 2:
*${\hat{M}}_{c}$ → AS:* *${\hat{M}}_{Ic}$ || ${\hat{M}}_{PKc}$
*
  *AS* →*${\hat{M}}_{c}$: $PK_{s}$*
  *${\hat{M}}_{c}$ → FS: $E_{PKs}$* (“Hello” ||${\hat{M}}_{PKc}$)
  $FS\to {\hat{M}}_{c}:E_{PKc}(K_{s,c}\left|\right|V_{s}\left|\right|\lambda_{s}\left|\right|PK_{s}^{{}^{\prime }}\left||ttl||mode=0\right)$.
  ${\hat{M}}_{c}$: cannot solve the ZKP challenge in the required time *ttl* for the default mode. Therefore, the protocol terminates;
- Severity Level 3:
  Mode 1: *${\hat{M}}_{c}$ → AS:${\hat{M}}_{Ic}$||${\hat{M}}_{PKc}$*
  *AS* →*${\hat{M}}_{c}$: $PK_{s}$*
  *${\hat{M}}_{c}$ → FS: $E_{PKs}$* (“Hello” ||${\hat{M}}_{PKc}$)

$FS\to {\hat{M}}_{c}:E_{PKc}(K_{s,c}\left|\right|V_{s}\left|\right|\lambda_{s}\left|\right|PK_{s}^{{}^{\prime }}\left||ttl||mode=1\right)$

${\hat{M}}_{c}↛FS:E_{PKs}^{{}^{\prime }}(K_{c,s}^{{}^{\prime }}\left|\right|V_{c}^{{}^{\prime }}\left|\right|\lambda_{c}^{{}^{\prime }}\left|\right|a_{c}^{{}^{\prime }})$

  Mode 2: *${\hat{M}}_{c}$ → AS:${\hat{M}}_{Ic}$||${\hat{M}}_{PKc}$*
  *AS* →*${\hat{M}}_{c}$: $PK_{s}$*
  *${\hat{M}}_{c}$ → FS: $E_{PKs}$* (“Hello” ||${\hat{M}}_{PKc}$)
  $FS\to {\hat{M}}_{c}:E_{PKc}(K_{s,c}\left|\right|V_{s}\left|\right|\lambda_{s}\left|\right|PK_{s}^{{}^{\prime }}\left||ttl||mode=2\right)$.

${\hat{M}}_{c}↛FS:E_{PKs}^{{}^{\prime }}(K_{c,s}^{{}^{\prime }}\left|\right|V_{c}^{{}^{\prime }}\left|\right|\lambda_{c}^{{}^{\prime }}\left|\right|a_{c}^{{}^{\prime }})$

  ${\hat{M}}_{c}$: cannot solve the above ZKP challenge in Mode 1 or 2. Therefore, the protocol terminates.

## 6. Protocol Verification Logic Analysis

To verify the protocol’s working in detail, the following logic analysis is presented for the hash-chain fog/edge protocol [41]:

### 6.1. Message Exchange

The message exchange between the client and server can be given as:

*A→S: A, $K_{a}$*

*S→A: $K_{b}$*

*A→B:* {$K_{a}$}${}_{K_{b}}$

$B\to A:{\left\{K_{ba},V_{b},\lambda_{c},K_{ab}^{{}^{\prime }},T_{l},Mode\right\}}_{K_{a}}$

$A\to B:{\left\{K_{ab},V_{a},\lambda_{a},P_{a}\right\}}_{K_{ab}^{{}^{\prime }}}$

$B\to A:{\left\{K_{ba},V_{b+1},V_{f}\right\}}_{K_{ab}^{{}^{\prime }}}$

$K_{ba}$ and $K_{ab}$ are shared session keys. $K_{ab}^{{}^{\prime }}$ are temporal session keys used in the subsequent communication for cryptography purposes. In the first message, the public key of client A is sent, which is verified and responded to by server S with a public key of fog server B. Later, B sends a challenge in the form of a temporal session key for the subsequent process. The challenge–response achievement then marks the successful completion of the protocol.

### 6.2. Idealized Protocol

The idealized protocol as per the rules can be stated as:

$A\to S:\overset{K_{a}}{⟶}A$

$S\to A:\overset{K_{b}}{⟶}B$

$A\to B:\{{\overset{K_{b}}{⟶}B\}}_{K_{b}}$

$B\to A:{\left\{A\overset{K_{\mathrm{ba}}}{⟷}B,V_{b},\lambda_{c},A\overset{K_{\mathrm{ab}}^{{}^{\prime }}}{⟷}B,T_{l},Mode\right\}}_{K_{a}}$

$A\to B:{\left\{A\overset{K_{\mathrm{ab}}}{⟷}B,V_{a},\lambda_{a},P_{a}\right\}}_{K_{ab}^{{}^{\prime }}}$

$B\to A:{\left\{A\overset{K_{\mathrm{ba}}}{⟷}B,V_{b},V_{f}\right\}}_{K_{ab}^{{}^{\prime }}}$

The idealized protocol is quite similar to the message exchange. It can be noticed that only entities and their respective known public keys are highlighted.

The public keys $K_{a}$, $K_{b}$ are shown in Messages 1, 2, and 3. Messages 4, 5, and 6 exchange public keys for confirmation with temporal session keys. The temporal session keys $K_{ab}^{{}^{\prime }}$ are then used for the cryptography operations of Messages 5 and 6.

### 6.3. Protocol Analysis

The protocol analysis as per the formal logic is constructed as:

$Abelieves\overset{K_{a}}{⟶}A$,

$Bbelieves\overset{K_{b}}{⟶}B$

$Abelieves\overset{K_{s}}{⟶}S$

$Bbelieves\overset{K_{s}}{⟶}S$

$Sbelieves\overset{K_{a}}{⟶}A$

$Sbelieves\overset{K_{b}}{⟶}B$

$Sbelieves\overset{K_{s}}{⟶}S$

$Abelieves(Scontrols\overset{K}{⟶}B)$

$Bbelieves(Scontrols\overset{K}{⟶}A)$

$Abelievesfresh(K_{ab})$

$Bbelievesfresh(K_{ba})$

$AbelievesA\overset{K_{\mathrm{ab}}}{⟷}B$

$BbelievesA\overset{K_{\mathrm{ba}}}{⟷}B$

$Bbelievesfresh(K_{ab}^{{}^{\prime }})$

The protocol analysis shows that all entities have their own public keys. Furthermore, entities A and B believe that server S knows their public keys for verification. Server S believes that A and B know their respective public keys. A and B trust S to invent good keys to control other public keys. A and B believe that they possess a fresh copy of the shared session keys for every new authentication. A and B believe that the fresh copy is known to each. B believes that it uses a fresh temporal session key ($K_{ab}^{{}^{\prime }}$) for the exchange of Messages 4, 5, and 6 for cryptography.

### 6.4. Final Beliefs

The final evaluation for the formal analysis can concluded as:

$Abelieves\overset{K_{b}}{⟶}B$

$Bbelieves\overset{K_{a}}{⟶}A$

$AbelievesA\overset{K_{\mathrm{ab}}^{{}^{\prime }}}{⟷}B$

$BbelievesA\overset{K_{\mathrm{ab}}^{{}^{\prime }}}{⟷}B$

$AbelievesBbelievesA\overset{K_{\mathrm{ab}}^{{}^{\prime }}}{⟷}B$

$BbelievesAbelievesA\overset{K_{\mathrm{ab}}^{{}^{\prime }}}{⟷}B$

In the final beliefs, both A and B believe that other entities have their own public keys. Later, the temporal session key ($K_{ab}^{{}^{\prime }}$) is common and shared between them. Both A and B believe that their respective counterpart knows the temporal session keys is shared between them. Once the counterpart beliefs are trusted, then the protocol is said to be secured and complete.

## 7. Results

In this section, we perform various experimental analyses to provide the details regarding the effectiveness of the hash-chain fog/edge protocol. The goal of this work is to present a detailed analysis of the protocol’s performance using independent session key generation analysis $K_{s,c}$, $K_{c,s}$, and updated $K_{s,c}$ comparison of the session key generation analysis results. All the session keys use different PRNG configurations, which are specified in detail below. Furthermore, these keys use SHA-256 bit hash [42] operations and the date-time format timestamp for more effective cryptographic operations.

### 7.1. System Configuration

Table 2 presents the system configuration deployed to perform the hash-chain fog/edge protocol experiments and Table 3 their respective supporting libraries. The configuration deploys a separate server, workstation, and Raspberry Pi with Ubuntu as the Linux operating system with Python as the programming language.

The Raspberry Pi has a size and cost similar to other low-end IoT devices. Thus, it is advantageous to use a low-power and low-cost device with a high processing power. For the AWS cloud, the Amazon Linux 2 Amazon Machine Image (AMI) Hardware Virtual Machine (HVM) was used for better compatibility. As discussed earlier, the key generation in different messages can be performed using different PRNG configurations for effective results. The PRNGs used are different types of linear congruential generators (LCGs), which are recommended as the best pseudo-random generators for cryptographic operations. This *PRNG* will be generated on the server-side *FS* and will have a performance as discussed in our previous work on SMAP fog/edge [43] in the Experiments Section.

### 7.2. Performance Analysis

**Figure 4:**
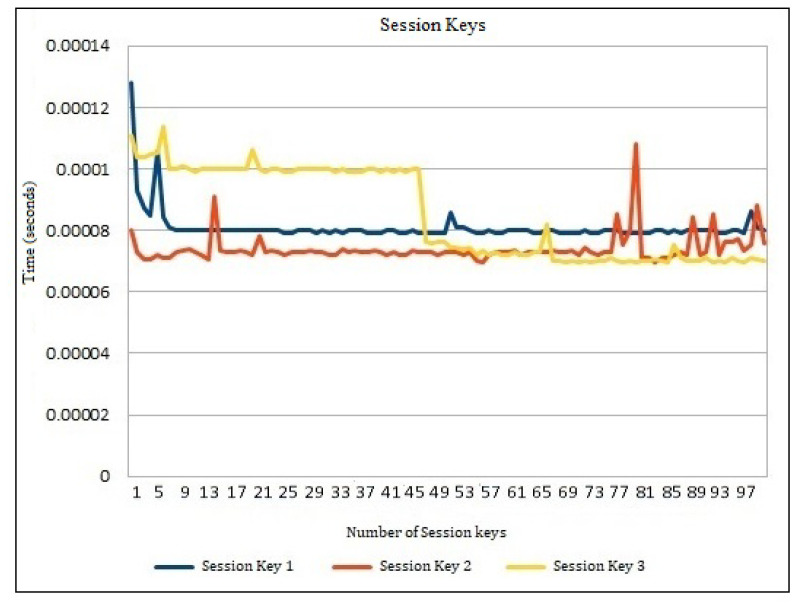
Time generation analysis of session keys.

**Figure 5:**
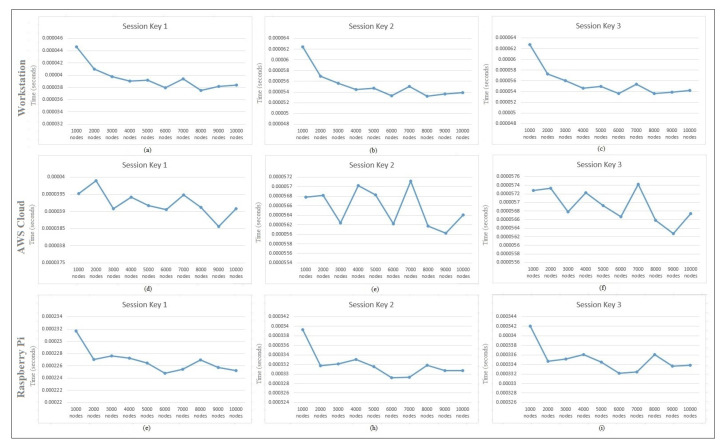
Session key generation time on the workstation for (**a**) Session Key 1, (**b**) Session Key 2, and (**c**) Session Key 3, the AWS Cloud for (**d**) Session Key 1, (**e**) Session Key 2, and (**f**) Session Key 3, and the Raspberry Pi (**g**) Session Key 1, (**h**) Session Key 2, and (**i**) Session Key 3.

Table 4 shows the performance analysis of various session keys generated during the hash-chain fog/edge protocol experiments. These keys present the independent time analysis for each key time required to be generated in the protocol message. It can thus be predicted that millions of keys can be generated in several fogs using a parallel working environment to authenticate several devices within the university/organization. Figure 4 presents the time generation analysis of session keys $K_{s,c}$, $K_{c,s}$, and updated $K_{s,c}$. It shows the detailed time generation of every key separately. These keys are calculated based on $K_{s,c}=H\left(T_{s}⊕\lambda_{s}\right),K_{c,s}=H\left(T_{c}⊕\lambda_{c}\right)$, and updated $K_{s,c}=H\left(T_{s}⊕\lambda_{s}\right)$. Figure 4 shows the performance comparison of session keys $K_{s,c}$, $K_{c,s}$, and updated $K_{s,c}$. No major difference was observed within the session key time generation comparison, and it was also found to be effective for several operations on the workstation. Figure 5 shows the session key generation time on the workstation, AWS Cloud, and Raspberry Pi each for Session Key 1, Session Key 2, and Session Key 3 in detail. These keys are presented to be compared in detail for the three different platforms. The cloud session key generation time was slightly faster as compared to the workstation, whereas the Raspberry Pi had a much lower rate of performance due to the limited amount of configurations compared to the other platforms. Even though the cloud performed faster, still the communication time of the keys with the edge devices may suffer. Thus, a workstation can be recommended for better authentication performance as a fog server *FS*. A similar performance can still be achieved with the Raspberry Pi when the fog network consists of only IoT devices.

**Figure 6:**
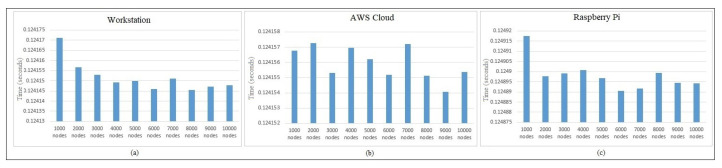
Independent protocol performance time on the (**a**) workstation, (**b**) AWS Cloud, and (**c**) Raspberry Pi.

**Figure 7:**
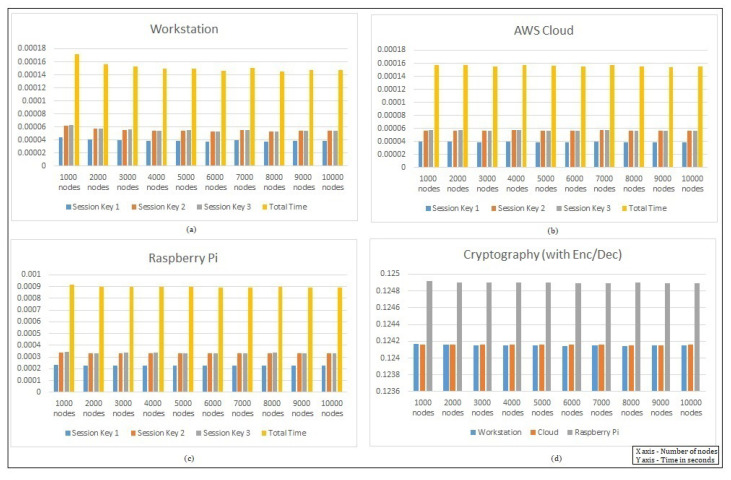
Performance with respect to the hash-chain fog/edge protocol total time on the (**a**) workstation, (**b**) AWS Cloud, and (**c**) Raspberry Pi and (**d**) for message exchange with cryptography.

Figure 6 presents the independent protocol performance times. As discussed earlier, the behavior across the architecture varies, and we can notice from the figure that, in the case of the workstation and Raspberry Pi, the total protocol time was quite similar and was seen to be adapting as the number of authenticating nodes increased. In the case of the cloud, the protocol authenticating time was seen to be quite balanced, as the execution was reinitialized across the infrastructure. In Figure 7, we checked the hash-chain fog/edge protocol total key generation time and total performance on different platforms for its analysis. The purpose of the experiments on different architectures represents the actual performance of the hash-chain protocol in the fog/edge network. In Part (a) for the workstation, we can see that the first session key $K_{s,c}$ required less time for the calculation of the first block of the hash chain process, whereas for the second session key $K_{c,s}$ and updated third session key $K_{s,c}$, the time required was similar, as they contained the hash from the previous blocks. The total time is a combination of all keys or the completion time for the whole protocol. In Part (b) for the AWS Cloud, the protocol followed a similar behavior as that of the workstation, including that of the session keys. In Part (c) for the Raspberry Pi, even though the behavior was the same as that of the workstation and cloud, the timing requirements were quite higher for all session keys and the total completion time for the protocol. In Part (d), the protocol message exchange completion time performed with cryptography (encryption/decryption) showed a similar behavior as that of the workstation and cloud even though the average key generation time fluctuated in different ranges. For the Raspberry Pi general-purpose computer, the protocol completion time was slightly higher because of the limited system configuration.

**Figure 8:**
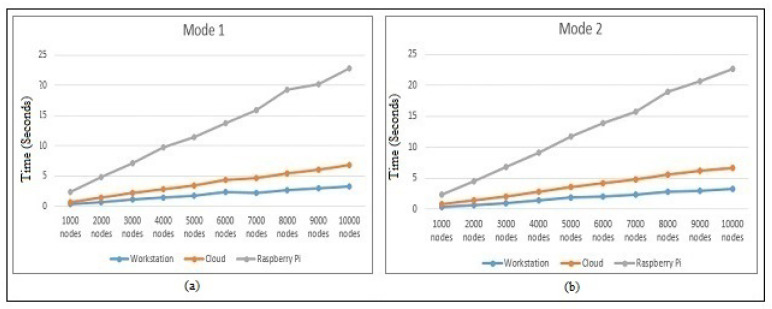
Performance with respect to the hash-chain fog/edge protocol in (**a**) Mode 1 and (**b**) Mode 2 time on the workstation, AWS Cloud, and Raspberry Pi.

Figure 8 demonstrates the hash-chain protocol performance’s in Mode 1 and Mode 2. On the workstation, cloud, and Raspberry Pi architectures, the time required for calculating Mode 1 and Mode 2 from Equation (1) and Equation (2) are shown, respectively. The workstation performed slightly better than the cloud, whereas the Raspberry Pi suffered (approximately by five-times) in calculating the protocol authentication process in different modes because of the large random values used for calculating the matrix inverse, transpose, and multiplication. The time required, as shown, was quite higher because the scenario considered all the authenticating devices entering the Mode 1 and Mode 2 challenge in the worst case. Regardless, such a scenario is quite rare, but still, we had to calculate it, for a detailed performance analysis.

Table 5 shows the comparison of the protocol objectives. Many recent protocols performing mutual authentication use session keys and hashes, such as Bansal et al. [14], Kumari et al. [26], Lopes et al. [27], Binu et al. [15], Wu et al. [29], and Madhusudhan et al. [30]. A higher cryptographic calculation was used in Bansal et al. [14], Kumari et al. [26], Lopes et al. [27], Binu et al. [15], Mbarek et al. [28], and Madhusudhan et al. [30]. Furthermore, active and advanced attacks were analyzed by all of them. While it appears that Kumari et al. [26] and Mbarek et al. [28] had shortcomings with respect to passive attacks and providing protocol verification logic, the detailed attack severity level was only provided by Kumari et al. [26] before the hash-chain. Ultimately, the hash-chain was found to complete with better logic of zero-knowledge proofs (ZKPs).

Table 6 shows the comparison of the 5G-AKA protocol [16,44] with the hash-chain fog/edge protocol, given its respective merit. One of our motivations was to overcome the problems faced by 5G security and design a system suitable for 5G communications and its peers, for future purposes. 5G suffers from secret key sharing with SN and HN, the repetition of keys (incrementing value) for authentication, a lack of cryptography, and channel attacks.

Table 7 shows the comparison of the computational cost for some of the protocols that were found to be relevant. $T_{Hash}$ denotes the time required to calculate the one-way hash function, $T_{E/D}$ the running time for the encryption/decryption of cryptographic operation, and $T_{ZKP}$ the time required to calculate the zero-knowledge-proof-based challenge–response operation for authentication. It can be seen that the hash-chain protocol performed better in comparison. A telecare medical information system that utilized a lightweight authentication protocol was demonstrated by Amin et al. [45]. The objective of this research work was anonymity preservation for the remote patient communication with the hospital by using a smart card. The entities involved were the home medical server, foreign medical server, physician server, and patient. The protocol involves multichannel connection and is mostly dependent on the hash of group identities for the protocol authentication. In wireless sensor networks, a real-time data access scheme using a lightweight three-factor scheme was implemented by Luo et al. [46]. A three-factor authentication scheme including biometrics, passwords, and smart cards was implemented between the entities of the user, gateway node, and sensor node. Twelve criteria were satisfied within this scheme with the confirmation of the fuzzy extractor. The authentication of remote users with key agreement was presented by Kumari et al. [47]. This scheme involves three entities as the user, smart card, and server, which can help update the passwords on the smart card. This scheme is usually operated by a combination of the timestamp, identity, passwords, and random numbers. It can be noticed that most of the protocols used only hash operations, which are quite vulnerable. The hash-chain performs better when authentication is required for a large number of devices because of the channel encryption. In Table 8, it can be seen that the hash-chain protocol still achieved better performance than the traditional RSA [40] algorithm with respect to the time requirements on Contiki OS, Cooja with Rime–Tmote Sky @ 3.9 MHz. In Table 9, an Ethernet comparison of hash-chain fog/edge protocol performance is shown. First, the workstation as a server authenticates the Raspberry Pi as the client. In the second part, the Raspberry Pi as a server is connected to authenticate another Raspberry Pi as the client. As both of the Ethernet connections have configurations as in Table 9, it can be noticed that due to the high computing power of the workstation, the protocol completion time without encryption/decryption was much less than the Raspberry Pi counterpart.

## 8. Conclusions

Successfully achieving all the objectives of the hash-chain fog/edge protocol of the distributed identity of key management, using a novel hash-chain algorithm, applying the zero-knowledge proof property for its operations, and the use of a minimum infrastructure with less calculation on IoT devices were demonstrated. The experimental results on different architectures, a workstation, the AWS Cloud, a Raspberry Pi, and the Cooja Simulator, and between their respective inter-connections showed its consistent behavior with similar time performance across different architectures while proving to be efficient. The paper showed that fog/edge operations utilize different types of architectures for their authentication and functioning. The protocol verification proof presented within the paper also provided its correctness. The computational cost comparison showed that the hash-chain performed better in the authentication for a higher number of devices. In future work, we would like to apply range proofs to experiment with different architecture combinations.

## Figures and Tables

**Table 1:** Comparison with the recent approaches.

References	Mutual Authentication Scheme	Entity’s Involved in the Authentication Process	Cryptography (Enc/Dec) or Message Communication	Protocol Implementation Scenario
Vehicle-to-grid (V2G) [14]	Physical unclonable function (PUF)-based secure user key exchange authentication (SUKA)	Vehicle, aggregator, and grid server	A function designed to perform XOR, addition, scalar multiplication, and exponential computation	A vehicle smart grid ecosystem (V2G)
Remote health monitoring [15]	A signature-based two-factor authentication protocol	The body sensors, personal devices (PDs), the medical server (MS), and the user (doctor/family)	A function using a secret key, a prime number, a generator of the cyclic group, a pseudo-random value, a hash function, an XOR operation, a concatenation operation, and nonce values	Remote health monitoring
5G security [16]	A signature-based mutual authentication protocol for m-health systems, which supports D2D communication within the 3GPP infrastructure	A health center, a cloud server, patients with and without sensors, patients’ devices, doctors, 3GPP access technology, evolved node B (eNB), and the 3GPP evolved packet core (EPC), represented by the home subscriber server (HSS)	Symmetric key with a random number, bi-linear pairing, and a signature	Mobile health (m-health) and telecare medicine information systems (TMISs)
Public key infrastructure (PKI)-IoT [11]	Enhanced elliptic-curve-cryptography (ECC)-based two-factor authentication framework	A user/smart card and server	Elliptic curve discrete logarithms problem (ECDLP) and elliptic curve computational Diffie–Hellman problem (ECCDHP)	Smart card authentication
Hash-chain fog/edge	A novel mode-based hash-chain mutual authentication protocol	A cloud server, a university gateway server, a department server, and a user/device	Symmetric key with the lightweight encryption system (LES)	A fog/edge model for inter-university student authentication

**Table 2:** Experimental setup.

System Environment	Server, Workstation	AWS Cloud	Raspberry Pi (3B+)
System Hardware	Intel Core i5 @ 3.10 GHz	T2.micro @ 2.5 GHz	Arm v8 @ 1.4 GHz
Primary Memory	16 GB	1 GiB	1 GB SDRAM
Operating System	Ubuntu 16.04	Amazon Linux 2 AMI	Ubuntu Server 19

**Table 3:** Libraries used in the different systems.

System	Library
AWS Cloud/Workstation (Server)	Random, hashlib, datetime and numpy.
Raspberry Pi	Random, hashlib, datetime, socket and JSON.
Contiki Cooja Simulator	<stdio.h>, <stdlib.h>, <string.h>, “contiki.h”, “net/rime.h”, “lib/list.h”, “lib/memb.h”, “dev/button-sensor.h” and “dev/leds.h”.

**Table 4:** Session key performance analysis.

Session Key	Generation Time in s (100 Keys Each)
** $K_{s,c}$ **	0.0080812
** $K_{c,s}$ **	0.00739694
Updated $K_{s,c}$	0.00851083

**Table 5:** Comparison of protocol objectives.

Features	[14]	[26]	[27]	[15]	[28]	[29]	[30]	Hash-Chain
1. Mutual Authentication	✓	✓	✓	✓	✓	✓	✓	✓
2. Session Key	✓	✓	✓	✓		✓	✓	✓
3. Zero-Knowledge Proofs								✓
4. Cryptography (Enc/Dec)	✓	✓	✓	✓	✓		✓	✓
5. Message Integrity	✓	✓	✓	✓		✓	✓	✓
6. Protocol Verification Logic	✓		✓		✓	✓	✓	✓
7. Active Attacks	✓	✓	✓	✓	✓	✓	✓	✓
8. Passive Attacks	✓		✓		✓	✓	✓	✓
9. Advance Attacks	✓	✓	✓	✓	✓	✓	✓	✓
10. Attack Severity Level’s		✓						✓

**Table 6:** 5G AKA protocol comparison.

Features	5G-AKA	Hash-Chain
1. Secret Key Sharing	Shared SN and HN Key	Not Shared
2. Challenge–Response	Key Hash, Random Number, and Identity	Zero-Knowledge Proof
3. Authentication Process (AP)	Hash Comparison and Key Seed	Mode-Based Tree and Graph Transition
4. Key Sharing	Repetition	Unique
5. Cryptography	No	Yes
6. Entities Involved in AP	SN, HN and Subscriber	Target Device, AS
7. Channel Attacks	Sensitive S-SN to MITM by Passive/Active Attacks	Uses Time-Based Hash-Chain
8. Management of Key Database	Uses Roaming for HN Proxy Connectivity	Blockchain Distributed Ledger
9. Structure of System Model	Hash Function and Key Exchange	Novel Hash-Chain

**Table 7:** Comparison of the computational cost to authenticate 1000 nodes.

Protocols	Total Computational Cost	Time (s)
Amin et al. [45]	26 $T_{Hash}$/21 $T_{Hash}$	8.32/6.72
Luo et al. [46]	26 $T_{Hash}$	8.32
Kumari et al. [47]	18 $T_{Hash}$	5.76
Hash-Chain	6 $T_{Hash}$+8 $T_{E/D}$+2 $T_{ZKP}$	0.125

**Table 8:** Comparison of standard benchmarks.

Protocol	Completion Time (ms)
RSA	23,500
Hash-Chain Fog/Edge	20,787

**Table 9:** Ethernet comparison.

System	Completion Time (s)
WS (server) to Pi (client)	0.0023081
Pi (server) to Pi (client)	0.0073781

## Notations

The following abbreviations are used in this manuscript:

*C*	Client/edge user
*S/FS*	Server/fog server
*AS*	Authentication server
$a_{x}$	Network IP address of x
*ttl*	Time to live/validity time of the message
$t_{x}$	Timestamp of x
$PK_{x}$	Public key of x
$SK_{x}$	Private key of x
$\lambda_{x}$	Transition value taken by x
$V_{x}$	Vertex taken by x
$R_{x}$	Random number of x
*M*	Message
$I_{x}$	Identity of x
$E_{x}$	Encryption by x’s public key
$D_{x}$	Decryption by x’s private key
$K_{x,y}$	Session key from x to y
$AS_{x,y}$	Authentication from x to y
${\hat{M}}_{x}$	Malicious user x
⊕	XOR bitwise operator
*X → Y*	X computes and sends to Y/transition operation

## Appendix A

### Appendix A.1. Details of the Registration Phase

The HCFE protocol addresses the open challenge of “Authentication and Key Agreement“ [3]. The management of keys was considered within the HCFE protocol distributed database (Section 3) where a potential application can be the cloud/the blockchain/a group of private servers. If we consider the blockchain, then the digital identity (ID) associated with the node is usually a decentralized public key. This public key is maintained at the top layer as per Figure 1 (Section 3.2). The registration phase is usually handled by the university admission office, which adds the new students upon successful admission, provides them a new identity (ID), and validates them. A parameter can be maintained within the public key database about the valid ID as the current student and the invalid ID as a graduate/withdraw/dropout/break/transfer student. Therefore, a new ID is stored within the distributed database, which can be later accessed through respective university gateways within different fog servers for the (Section 3.1) validation of a user/client visiting different universities within the country and obtain authentication with the help of the hash-chain fog/edge (HCFE) Protocol (Section 3.3). The user/client is successfully authenticated only in the case of solving the challenge model based on the (Section 1.1) zero-knowledge proof (ZKP) properties of completeness, soundness, and zero-knowledge.

### Appendix A.2. Description of the Attacks

As referenced in the description of the attacks in our previous paper on SMAP fog/edge [43], all the active, passive, and advanced attacks’ descriptions were given. The purpose behind using the different attack scenarios was to present to the readers complete knowledge of how the HCFE protocol is well equipped even in the face of security, as given in Section 5, against active, passive, and advanced attacks. The sinkhole attack, even though it takes place during the routing of network packets by advertising a fake routing update, still would be unable to work in its application of selective forwarding, as the challenge tree transitions, timestamp, and session keys would be unique every time. In the case of the active attack of modification, the message can be tampered with, resulting in integrity inconsistency; thus, changing any of the parameters makes the protocol step terminate the session. For the passive attack of eavesdropping, the message can be either intercepted, modified, or deleted, which will not be harmful as the hash values and encrypted messages will be made inconsistent, causing the session to terminate. As per the basic definition of the denial of service (DOS) attack, a target is flooded with high traffic, which simply will lead to terminating all the attempts made at the initial level of the HCFE protocol without causing any significant damage. The new advanced attack covered within HCFE is Sybil: when multiple peer identities are being counterfeited by a small number of entities, which compromises a disproportionate share of the system. As referenced in [16,36,37], the control of the Sybil attack can be achieved by the admission control mechanism for distributed databases, which periodically solves the computational puzzles. Nevertheless, all the security attacks presented within Section 5 were three different security levels, helping to improve readers understanding.

### Appendix A.3. Acronyms

Internet of Things (IoT), IoT distributed denial of service attack (IDDOS), hash-chain fog/edge (HCFE), zero-knowledge proof (ZKP), 5th-Generation Mobile Network (5G), distributed database system (DDS), pseudo random number generator (PRNG), ticket-granting server (TGS), authentication server (AS), Compute Unified Device Architecture (CUDA), intellectual property rights (IPR), National Health Insurance (NHI), wireless fidelity (WiFi), machine-to-machine (M2M), end user (EU), unmanned aerial vehicle (UAV), public key infrastructure (PKI), Datagram Transport Layer Security (DTLS), one-time password (OTP), identity-based elliptical curve cryptography (IBE-ECC), edge-disjoint path (EDP), nondeterministic polynomial-time complete (NP-complete), device-to-device (D2D), radio frequency identification (RFID), peer-to-peer (P2P), wireless sensor network (WSN), Amazon Web Services (AWS), 5G Authentication and Key Agreement (AKA), serving network (SN), home network (HN).
